# Human Primary Bone Marrow Mesenchymal Stromal Cells and Their *in vitro* Progenies Display Distinct Transcriptional Profile Signatures

**DOI:** 10.1038/s41598-017-09449-x

**Published:** 2017-09-04

**Authors:** Roshanak Ghazanfari, Dimitra Zacharaki, Hongzhe Li, Hooi Ching Lim, Shamit Soneji, Stefan Scheding

**Affiliations:** 1 Lund Stem Cell Center, University of Lund, Lund, Sweden; 2 Division of Molecular Hematology, Department of Laboratory Medicine, University of Lund, Lund, Sweden; 3 Department of Hematology, Skåne University Hospital Lund, Lund, Sweden

## Abstract

Bone marrow mesenchymal stromal cells (BM-MSCs) are a rare population of cells that gives rise to skeletal tissues and the hematopoietic stroma *in vivo*. Recently, we have demonstrated that BM-MSCs fulfill stringent *in vivo* stem cell criteria when propagated as non-adherent mesenspheres but not as adherent-cultured cells. Motivated by these profound functional differences, the current study aimed to identify potential important MSC regulators by investigating global gene expression profiles of adherent and non-adherent culture-derived BM-MSCs in comparison with primary BM-MSCs. A substantial number of genes were differentially expressed between primary and culture-expanded cells already early upon culture, and numerous genes were found to be different when comparing adherent and non-adherent BM-MSCs. Cluster analysis identified 16 sets of genes of which two displayed comparable gene expression levels in primary and non-adherent cultured cells, but not in adherent cultured cells. This pattern suggested that these clusters contained candidate regulators of BM-MSCs. Gene expression differences were confirmed for selected genes and BM-MSC transcription factors by protein analysis and RT-PCR, respectively. Taken together, these data demonstrated profound gene expression changes upon culture of primary BM-MSCs. Moreover, gene cluster differences provide the basis to uncover the regulatory mechanisms that control primary and cultured BM-MSCs.

## Introduction

Despite significant progress in bone marrow mesenchymal stromal cells (BM-MSCs) biology and the widespread clinical application of cultured BM-MSCs, uncertainties remain regarding the differences of culture-expanded cells and their primary “bona-fide” BM-MSC counterparts.

By tradition, BM-MSCs are identified retrospectively based on their typical capacity to adhere to plastic surfaces and form colonies *in vitro*. However, cultured BM-MSCs are a heterogeneous population^1^ with reduced multilineage differentiation potential^2^ and decreased hematopoietic support ability compared to primary cells, and they fail to home to the bone marrow following intravenous infusion^3^. Additionally, cultured BM-MSCs display a different phenotypic, genetic and epigenetic profile compared to freshly isolated primary cells^4, 5^. These reported functional and phenotypical differences highlight the importance of prospectively isolating primary cells when aiming to investigate the role of native BM-MSCs, but also suggest that biological differences between primary and cultured BM-MSCs might be the key to identify and understand basic important biological BM-MSC properties.

Identification and characterization of primary BM-MSCs has conventionally relied on prospective isolation usually by a combination of two or more surface markers. Reported markers for primary BM-MSCs include CD271 (LNGFR)^6^, CD106 (VCAM-1) and STRO-1^7^, CD146 (MCAM)^8^, CD90 (THY-1)^9^, CD105 (Endoglin)^10^, frizzled-9 and SSEA-4^11^, CD51 (ITGaV)^12^, CD140a (PDGFRa)^12^, SUSD2 (W5C5)^13^, MSCA-1^14^, CD230 (PrP)^15^ and LEPR^16^.

Previous work from our group demonstrated that the combination of CD271^+^/CD140a^low/negative^ allowed to identify a population of cells that was very highly enriched for clonogenic BM-MSC, i.e. CFU-F (colony-forming units, fibroblast)^17^. However, as also the CD140a^+^ fraction contained CFU-F potential - although at a much lower level compared to the PDGFRa^low/negative^ cells, we chose for the current study to apply the broader marker combination lin^−^/CD45^−^/CD31^−^/CD71^−^/CD235a^−^/CD271^+^ that selects for all bone marrow cells with CFU-F activity, thus allowing to better compare our data with traditional MSC enrichment approaches.

Traditional two-dimensional adherent cultures have been implicated in affecting cell polarity, shape, growth, morphogenesis, gene expression, motility and cell differentiation, amongst other aspects of cell biology^18^. Therefore research has focused during the past decade on functionally improving *in vitro* culture systems to resemble more the physiological state by introducing non-traditional three-dimensional culture systems, e.g. by using natural hydrogels, synthetic polymers and solid scaffolds^19^. Recently, we have shown that expansion of human BM-MSCs as non-adherent mesenspheres preserved their immature phenotype^20^, and, importantly, promoted their *in vivo* self-renewal capacity in serial transplantations^21^. These findings indicated an advantage of non-adherent sphere cultures over conventional adherent systems to preserve stem cell properties, which prompted us to investigate possible gene expression differences between prospectively-isolated primary BM-MSCs, adherent- and sphere-cultured BM-MSCs.

Utilizing gene expression array analysis, our current study clearly identified distinct clusters of differentially expressed genes in primary and cultured BM-MSCs. Profound gene expression differences were observed between primary and cultured cells, and differences were also present between adherent and sphere BM-MSCs. Gene expression changes over time, however, were less pronounced under both culture conditions. Furthermore, gene expression cluster analysis allowed us to identify potentially important BM-MSC regulators. The BM-MSC gene expression profiles reported herein thus provide the basis to identify the mechanisms that cause the observed functional differences of primary and cultured BM-MSCs.

## Results and Discussion

### Gene expression profiles differed considerably between primary and cultured bone marrow mesenchymal stromal cells

Although adherent-culture expanded BM-MSCs have been used in numerous studies and serve as attractive candidates for cell-based therapies, little is known about their phenotypic and functional relationship with their primary counterparts of which they are derived from. Additionally, phenotypic characteristics of sphere-cultured BM-MSCs in comparison to adherent-cultured and primary BM-MSCs have not been studied yet.

In the present study we therefore chose to use standard adherent cultures and novel non-adherent mesensphere culture methods for *in vitro* expansion of BM-MSCs, and compared the gene expression profiles of these cultured BM-MSCs with prospectively isolated primary bone marrow stromal cells.

**Figure 1:**
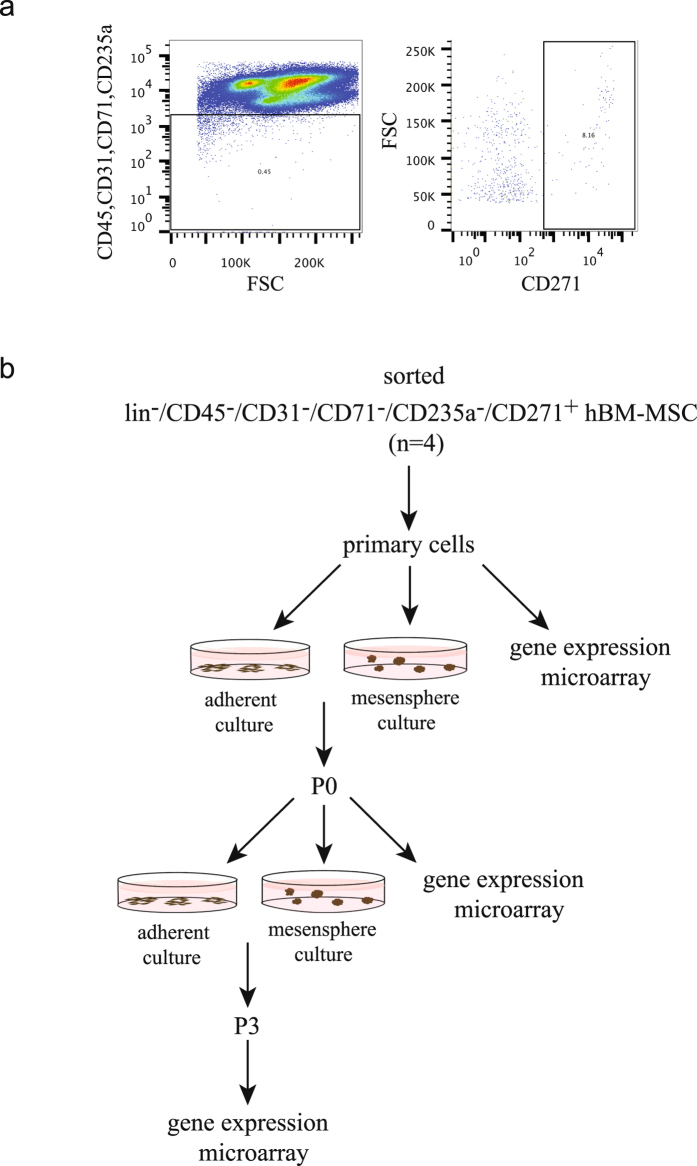
FACS gating strategy and experimental design of the microarray analysis. a. Freshly isolated, lineage-depleted bone marrow mononuclear cells were stained with antibodies against CD45, CD31, CD71, CD235a and CD271 as described. Following forward/side scatter gating and dead cell exclusion, CD45^−^/CD31^−^/CD71^−^/CD235a^−^ cells were sorted by gating on the CD271^+^ population. A representative set of FACS plots is presented. b. Schematic overview of the experimental workflow. From each donor (n = 4), primary cells were sorted into lysis buffer for gene expression analysis, and for culture in mesensphere and standard MSC medium, respectively. *Ex-vivo* cultured adherent BM-MSCs and mesenspheres were harvested in passages 0 and 3, and prepared for microarray analysis.

Several promising BM-MSC markers in combination with CD271 have been reported to enrich for fractions of BM-MSCs with high CFU-F content and potent hematopoietic support (for review, see ref. 22). However, the level of overlap between these markers has not been thoroughly resolved in terms of their spatial and functional contributions to the stroma compartment and the hematopoietic niche. The CD271 marker, although not specific for BM-MSCs, has been shown to detect all CFU-F in normal human bone marrow^23^ and was therefore used in combination with exclusion markers for hematopoietic and endothelial cells to isolate fresh bone marrow stromal cells (Fig. 1a, the experimental design is illustrated in Fig. 1b).

**Figure 2:**
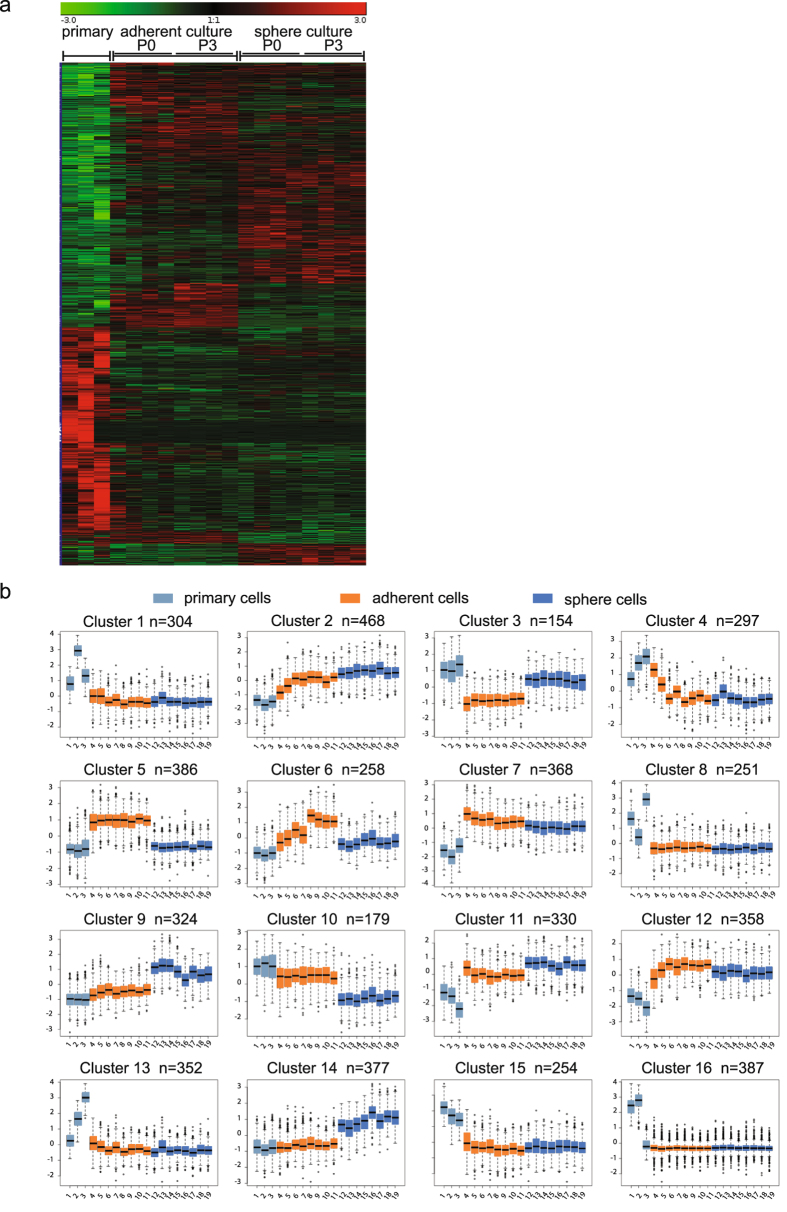
Gene expression and cluster analysis of primary, adherent and non-adherent culture-expanded BM-MSCs. (**a**) Heatmap of significantly differentially expressed genes in primary, adherent and sphere cultured-derived lin^−^/CD45^−^/CD31^−^/CD71^−^/CD235a^−^/CD271^+^ cells at passages 0 and 3. (**b**) Cluster analysis of differentially expressed genes. The first data points from the left in each plot (1–3, light blue) represent primary cells, the next 8 boxes in orange represent adherent cultured cells (4–7: passage 0; 8–11: passage 3) and the last 8 boxes in dark blue represent the results for sphere cultured cells (12–15: passage 0, 16–19: passage 3).

The gene expression profiles of primary lin^−^/CD45^−^/CD31^−^/CD71^−^/CD235^−^/CD271^+^ cells and BM-MSCs derived from these sorted primary cells in adherent and sphere cultures, respectively, are shown as a heatmap in Fig. 2a. In total, 5,047 genes showed significant differential expression levels among the three groups. Interestingly, gene expression in primary cells (left column) was clearly distinct from both cultured cell types. Approximately 1,900 genes were up-regulated and around 1,100 genes were down-regulated in primary cells compared to adherent and sphere cultured cells. In contrast, expression levels of considerably fewer genes in primary cells were similar to either adherent cultured BM-MSCs (middle column) or sphere-cultured cells (right column). Furthermore, a large number of genes were found different when comparing adherent BM-MSCs with sphere-cultured cells. Remarkably, increasing time in culture did not induce additional major gene expression changes in neither adherent nor sphere-cultured cells (Fig. 2a).

These findings clearly indicate that the main gene expression changes occurred directly when primary bona-fide BM-MSCs were taken into culture. To our knowledge, this aspect has not been reported thus far, but is certainly of importance when considering that culture-derived BM-MSCs have been studied previously to identify BM-MSC aberrations in hematological malignancies^24, 25^. Clearly, in these kinds of experiments, possible culture-induced gene expression changes have to be taken into account when aiming to deduce primary BM-MSC properties from data generated with cultured BM-MSCs. Preferably, experiments should therefore be performed directly on prospectively isolated primary cells, which however are very rare cells. Nevertheless, effective primary BM-MSC isolation has become possible utilizing advanced sorting and analysis methods as shown by us and others for normal bone marrow^3, 17^, and was also recently applied to study prospectively-isolated primary human BM-MSCs from MDS (myelodysplastic syndrome) patients^26^.

### Gene cluster analysis revealed distinct gene expression patterns in primary and cultured BM-MSCs

Based on the gene expression level differences among the three groups of cells (primary, adherent and sphere cultured cells), the data were assigned to 16 different clusters using the k-means algorithm (Fig. 2b) reflecting principally distinct expression patterns. Each cluster represented a certain number of genes with no overlap between the clusters.

Eight clusters showed upregulated (clusters 1, 8, 13, 15, 16) or downregulated (clusters 7, 11, 12) gene expression in primary BM-MSCs compared to both, adherent and sphere cultured cells, which exhibited similar gene expression levels for the genes in this cluster. These clusters thus represent culture-induced changes of primary cells, regardless of the culture method employed.

Other gene clusters showed similar expression levels in primary cells and in either the adherent cells (clusters 9, 10, 14) or the sphere cultured cells (clusters 3, 5, 6). Genes that were included in these clusters are therefore candidates not only to investigate culture-specific differences but also to identify possible regulators of primary BM-MSC properties that are preserved under one culture condition but lost when cells are cultured in the other culture system. An example for such a property is the *in vivo* serial transplantation capacity of BM-MSCs, which is preserved in spheres but not in adherent cultures^21^. Based on this finding, clusters 3 and 5 were analyzed separately and in more detail (see below).

On the other hand, cells propagated in both, adherent and mesensphere conditions maintained *in vitro* clonogenicity and tri-lineage differentiation potential^2^ suggesting that culture-induced expression changes of genes related to *in vitro* proliferation or *in vitro* multi-lineage differentiation genes are elusive to detection by conventional assays. This might be due to the artificial environment and induction system used to evaluate these *in vitro* properties which might not be suitable to detect any functional differences at early times in culture. Nonetheless, BM-MSC *in vitro* differentiation potentials have been reported to decrease in later passage cells^2^, which were not investigated in the current study but could be used to study differentiation-related gene expression changes in future experiments.

As mentioned above, it is also important to note that duration of culture, i.e. from short-term (passage 0) to long-term culture (passage 3) did not translate into additional profound gene expression profile changes, regardless of the culture type. This clearly indicates that the majority of changes occur early in culture.

Taken together, these data show the remarkable change of the genetic profile of primary BM-MSCs upon culture, even when exposed to culture conditions only for a short period of time. This finding thus clearly emphasizes the importance of studying biological and functional properties of BM-MSCs in their native state. Furthermore, our data suggest that possible functional alterations due to culture-induced genetic changes need to be taken into consideration when BM-MSCs are intended for therapeutic use.

### Detailed analysis of gene clusters showing similar expression levels in primary and sphere cultured cells but not in adherent cells

Among the 16 different clusters, numbers 3 and 5 showed similar gene expression levels in primary and sphere cultured cells, while they were clearly different in the adherent cultured cells. Thus, these clusters are likely to contain possible candidate genes that are involved in maintaining BM-MSC stem cell properties in sphere cultures, e.g. *in vivo* self-renewal capacity^21^. Therefore, these clusters were chosen for a more detailed analysis.

**Figure 3:**
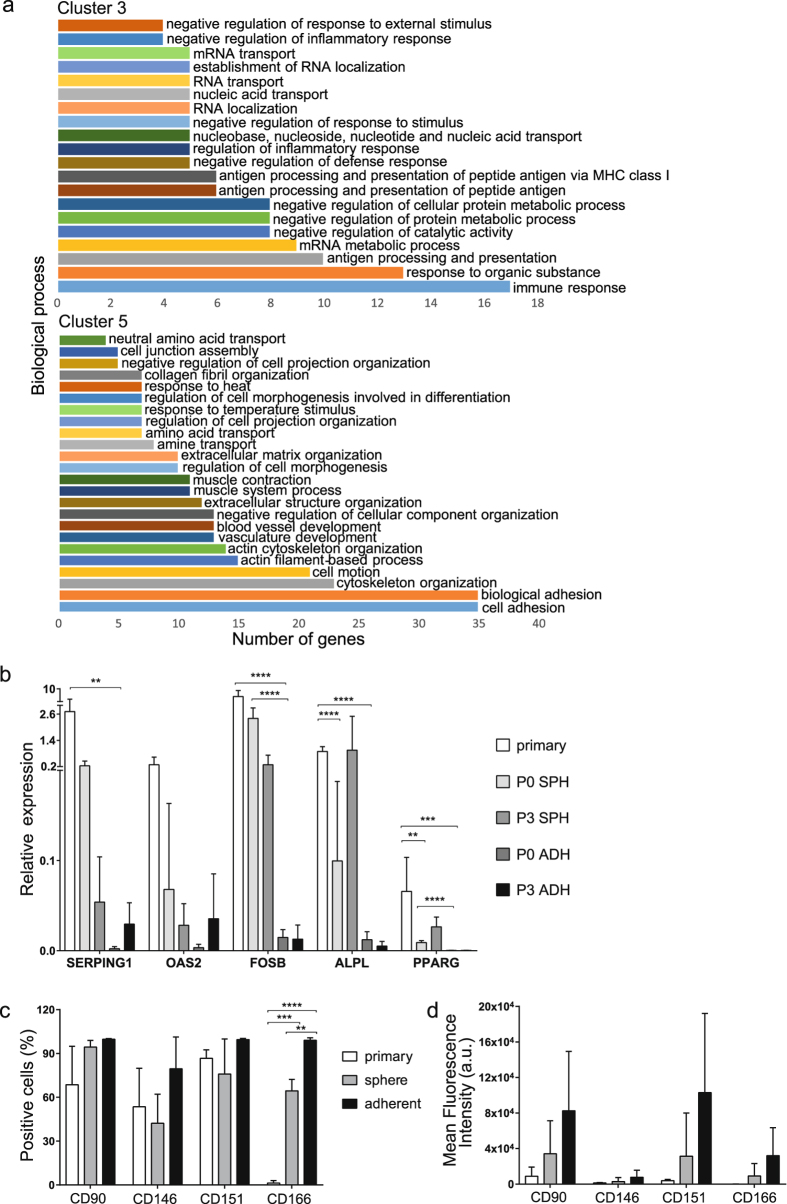
Primary and sphere-cultured cells but not adherent cultured cells show similar expression patterns in clusters 3 and 5. (**a**) Biological process annotations with false discovery rate (FDR) ≤ 0.1% in clusters 3 and 5 were identified using the DAVID Bioinformatics Resources 6.8. The x-axis indicates the number of genes included in each functional group. Cluster 3 shows the genes that were up-regulated in primary and sphere-cultured cells, but down-regulated in adherent-cultured cells. Cluster 5 contains genes that were down-regulated in primary and sphere-cultured cells but up-regulated in adherent-cultured cells. The names of the genes in clusters 3 and 5 are listed in Supplementary Table S1. (**b**) Gene expression analysis of cluster 3 genes relative to the RPL11 reference gene (n = 3). Significant differences in gene expression between primary and cultured cells (P0 and P3) are indicated as: **p < 0.01, ***p < 0.001, ****p ≤ 0.0001 (2-way ANOVA, multiple comparisons test). (**c**) Percentage of positive cells for each surface marker in primary, adherent and sphere cultured BM-MSCs (n = 3, 2-way ANOVA, p = 0.0005), and (**d**) Mean fluorescence intensity (geometric means) of each surface marker minus their respective isotype (n = 3, 2-way ANOVA p = 0.55, cell group variation p = 0.0149, surface marker variation p = 0.1027). Bars show mean ± SD. SPH: sphere cultured cells, ADH: adherent cultured cells.

Cluster 3 included a total of 154 genes that were upregulated in primary and sphere cultured cells but not in adherent cultured cells. In cluster 5 there were 386 genes which had a higher expression in adherent cells in comparison with primary and sphere cultured cells (Fig. 2b and Supplementary Table S1). Gene ontology analysis revealed that the differentially expressed genes could be assigned to a diverse range of biological processes in each cluster of genes (Fig. 3a). In cluster 3, most of the genes were involved in immune response, mRNA processes and antigen presentation. The expression of several genes belonging to cluster 3 such as SERPING1, involved in the regulation of the complement cascade, OAS2, reported in antitumoral immunity and ALPL, involved in osteogenesis, was additionally validated by quantitative real-time PCR (Fig. 3b). In contrast, cluster 5 consisted of genes mainly involved in cell adhesion, cytoskeleton organization, cell motion and vasculature development.

Next, to more specifically identify possible regulatory molecules relevant to BM-MSC properties, transcription factors were identified^27^ among the genes in clusters 3 and 5 (Table 1). Some of these transcription factors have been reported to play a role in the regulation of BM-MSCs or other stem cells, including: FOS, FOSB, HMGB3, EGR1, PPARG, ATF4, NFE2L1 and SOX4.

**Table 1:** Transcription factors expressed in cells in clusters 3 and 5.

Name	Description
**Transcription factors in cluster 3**	
FOS	Fos Proto-Oncogene, AP-1 Transcription Factor Subunit
FOSB	FosB Proto-Oncogene, AP-1 Transcription Factor Subunit
TBP	TATA-Box Binding Protein
HMGB3	High Mobility Group Box 3
RFX5	Regulatory Factor X, 5 (Influences HLA Class II Expression)
HLX	H2.0 Like Homeobox
EGR1	Early Growth Response 1
PPARG	Peroxisome Proliferator Activated Receptor Gamma
**Transcription factors in cluster 5**	
FOXD1	Forkhead Box D1
TULP4	Tubby Like Protein 4
ATF4	Activating Transcription Factor 4
NFE2L1	Nuclear Factor, Erythroid 2 Like 1
ZFHX3	Zinc Finger Homeobox 3
ID3	Inhibitor of DNA Binding 3
ID2	Inhibitor of DNA Binding 2
ATOH8	Atonal BHLH Transcription Factor 8
CREB3L2	CAMP Responsive Element Binding Protein 3 Like 2
SOX4	SRY (Sex Determining Region Y)-Box 4
ZNF197	Zinc Finger Protein 197

Cluster 3 genes FOS and FOSB are members of the AP-1 transcription factor family, known to be involved in osteoblast differentiation^28^. Expression of FOS and FOSB has furthermore been reported to be induced in human mesenchymal stromal cells following mechanical stress and leads to activation of osteogenic differentiation^29, 30^. Quantitative real-time PCR showed that FOSB was indeed highly expressed in primary cells whereas its expression was reduced in sphere cultured cells (by 4-fold) and dramatically reduced by > 10-fold in adherent cultured cells (Fig. 3b).

PPARG, also present in Cluster 3, is a gene that serves as the main regulator of adipogenesis, which, in concert with other transcription factors such as C/EBP molecules, induces adipogenic differentiation^31^. PPARG expression was furthermore found to be high in primary cells compared to either culture condition and showed significant differences between P0 adherent and P0 sphere cultured cells (Fig. 3b). The overall pattern of expression is in accordance with cluster 3 results, i.e. low expression in adherent cells compared to both primary and sphere cultured cells (Fig. 3b).

HMGB3, another cluster 3 gene, encodes a DNA binding protein and maintains the hematopoietic stem cell (HSC) population by regulating the balance between self-renewal and differentiation^32^. However, its possible role in stromal stem cells is currently unknown and HMGB3 is therefore certainly a candidate for future experiments. EGR1 is another cluster 3 transcription factor involved in a number of different cellular functions. The role of EGR1 in HSCs has been previously reported^33^ and, importantly, own recent data showed that EGR1 expression negatively regulated BM-MSCs proliferation and colony formation but positively regulated hematopoietic stroma support function (unpublished data). Thus, EGR1 is a likely key regulator of human primary stroma cells and experiments are ongoing to identify the downstream targets of EGR1 that are relevant for hematopoietic support.

Cluster 5 contained a total of 11 transcription factor genes. Of these, ATF4 and NFE2L1 are involved in osteogenesis. ATF4 promotes osteoblast differentiation of BM-MSCs through different mechanisms, including activation of the Osteocalcin gene, the Osterix gene, and by increasing the protein levels of β-catenin^34^. NFE2L1 is expressed by osteoblasts and induces osteogenesis through activation of Osterix^35^. SOX4 has a key role in cell fate decisions during the embryonic period and is furthermore a major regulator of epithelial-mesenchymal transition (EMT)^36^.

Based on these known functions of the identified transcription factors, these genes can thus be considered as candidates for future studies utilizing knock-down or overexpression approaches to investigate their regulatory roles in proliferation, differentiation and hematopoietic support of native primary BM-MSCs. ChIP sequencing could then be applied to determine the DNA binding sites of candidate transcription factors.

Our study focused on transcriptional differences identified between primary BM-MSCs and BM-MSCs cultured both as 2D adherent cells and 3D mesenspheres. In order to assess whether protein levels followed the pattern of gene expression as shown in our microarray data we evaluated the surface marker expression of four selected genes that have been reported in the context of MSC function and differentiation, i.e. CD90 (Thy-1), CD146 (MCAM), CD151 (GP27) and CD166 (ALCAM) in primary BM samples and passage 3 (P3) adherent cultured and sphere-cultured cells. Here, flow cytometry analysis showed that primary cells were different from cultured cells with regard to surface marker expression, both in terms of the number of positive cells (Fig. 3c, and Supplementary Fig. S1), and mean fluorescence intensities (Fig. 3d), which furthermore reflected the microarray data pattern (Supplementary Table S1). Additionally, we investigated the expression of CD105 as key BM-MSC marker, which was positive on all cell preparations (data not shown).

In summary, gene expression analysis of primary and cultured BM-MSCs showed a significant change of the molecular signature of these cells upon culture. Moreover, cluster analysis allowed to identify potentially important BM-MSC regulators. These data therefore provide the basis for future studies aiming to gain insight into gene regulation mechanisms that play an important role in different biological processes of the human stroma cell compartment.

## Materials and Methods

### Bone marrow mononuclear cells (BM-MNCs)

Bone marrow samples were aspirated from the iliac crest of healthy donors (60 ml, n = 21, median age 25, range 18–45). All participants in the study gave a written informed consent. The procedure was approved by the Swedish local ethics committee in Lund (Regionala Etikprövningsnämden Lund, EPN, protocol Dnr2009/532) and all experimental protocols were performed according to EPN’s guidelines. Bone marrow mononuclear cells (BM-MNCs) were isolated by density gradient centrifugation (Ficoll-paque medium, GE Healthcare Life Sciences) combined with prior incubation with RosetteSep Human Mesenchymal Stem Cell Enrichment Cocktail (StemCell Technologies) for lineage depletion (CD3, CD14, CD19, CD38, CD66b and glycophorin A).

### Fluorescence activated cell sorting (FACS)

Lineage-depleted BM-MNCs were incubated in blocking buffer [DPBS w/o Ca^2^^+^, Mg^2^^+^, 3.3 mg/ml human normal immunoglobulin (Octapharma) and 1% fetal bovine serum (Life Technologies)], followed by staining with monoclonal antibodies against CD45-FITC (clone 2D1), CD31-FITC (clone WM59), CD71-FITC (clone M-A712), CD235a-FITC (clone GA-R2), CD90-PE (clone 5E10), CD146-PE (clone P1H12), CD151-PE (clone 14A2.H1), and CD166-PE (clone 3A6) all from BD Biosciences and CD271-APC (clone ME20.4–1.H4, Miltenyi Biotec). Sorting and analysis gates were set according to the corresponding fluorescence-minus-one (FMO) controls. Cells were sorted on a FACS Aria II or a FACS Aria III cell sorter (BD Biosciences) or analysed on an LSRII flow cytometer (BD Biosciences). Dead cells were excluded by 7-amino-actinomycin (7-AAD, Sigma) staining, and doublets were excluded by gating on FSC-H vs FSC-W and SSC-H vs SSC-W. The cells were sorted either into RLT lysis buffer (RLT buffer plus 1% β-ME) provided in the RNeasy Plus Micro Kit (Qiagen) or into MSC culture medium (StemMACS MSC Expansion Medium [Miltenyi Biotec] plus 1% antibiotic-antimycotic solution [Sigma]) and sphere growth medium^37^ for adherent and non-adherent expansion, respectively. The FlowJo (v 10.1, Tree Star Inc.) software package was used to analyze the data, and geometric means (mean fluorescence intensity, arbitrary units) were determined as previously described^38^.

### Generation of adherent marrow stromal cells

Sorted BM-MNCs were cultured in standard MSC culture medium. Medium was changed weekly and cells were passaged as described^23^. A portion of cells at passage zero was transferred to lysis buffer for further analysis. The remaining cells were sub-cultured and replated at 500-1,000 cells/cm^2^ until passage three when they were harvested and lysed in the buffer for RNA analysis.

### Non-adherent stroma cell cultures (mesenspheres)

Sorted BM-MNCs were plated at low density ( < 1,000 cells/cm^2^) in ultra-low adherence plates (Corning) in sphere growth medium as described before^37^. The medium contained 15% chicken embryo extract (CEE), 0.1 mM β-mercaptoethanol (Sigma), 1% non-essential amino acids, 1% antibiotic-antimycotic solution (Sigma), 1% N2 supplement, 2% B27 supplement (both from Invitrogen), recombinant human oncostatin (20 ng/ml, Invitrogen), recombinant human epidermal growth factor (EGF, 20 ng/ml, Invitrogen), recombinant human fibroblast growth factor (FGF)-basic (20 ng/ml, Invitrogen), recombinant human platelet-derived growth factor (PDGF-AB) (20 ng/ml, Peprotech) and recombinant human insulin-like growth factor-1 (IGF-1) (40 ng/ml, Invitrogen) in DMEM/F12 (1:1)/human endothelial serum-free medium (1:2) (Invitrogen). CEE was prepared as described previously^39^. To prevent cell aggregation, cultures were left untouched for 1 week. Thereafter, half-medium changes were performed twice weekly. Spheres were passaged following enzymatic digestion with 0.25% type I collagenase (StemCell Technologies) for 30 min at 37 °C, followed by washing with PBS, and re-plating at clonal density.

### Real-time quantitative PCR

RNA from FACS-sorted CD271^+^cells, P0 and P3 adherent and sphere cultured cells (n = 3 per cell group, the same donors were used in adherent and sphere cultures) was isolated using RNeasy Micro Kit (Qiagen) following the manufacturer’s instructions. Complementary DNA was synthesized using the Superscript Vilo cDNA synthesis kit (Invitrogen). Our microarray data showed that commonly used housekeeping genes, such as GAPDH and BM2, were differentially expressed among primary, adherent BM-MSC and sphere cultured BM-MSC. Therefore we used RPL11 as reference gene, which was expressed at similar and constant levels across the three conditions in the microarray. The primer pairs used are listed in Supplementary Table S2. Reactions were set up in duplicates using Fast SYBR master mix (Applied Biosystems) and run in three independent experiments. Data were analyzed with StepOne software (v2.3, Applied Biosystems) based on the ΔCt method.

### Statistical analysis

Grouped data were analyzed using two-way analysis of variance (2-way ANOVA) and multiple comparisons across samples were performed using two-stage linear step-up procedure of Benjamini, Krieger and Yekutieli, with Q = 1% in GraphPad Prism v7 (GraphPad Software Inc.). The significance level was set at α = 0.05. All data are shown as mean ± SD from at least 3 independent experiments unless stated otherwise.

### Microarray Expression Analysis

Total RNA from primary sorted lin^−^/CD45^−^/CD31^−^/CD71^−^/CD235a^−^/CD271^+^ cells, and adherent and sphere culture-generated passage 0 and passage 3 cells, respectively, were isolated using the RNeasy plus micro-kit (Qiagen) according to the manufacturer’s instructions. In three out of four cases, samples were donor-matched across primary, adherent and sphere cultured cells, and all adherent and sphere cultured cells were donor-matched across all passages. RNA was subjected to a two-round amplification using the TargetAmp^TM^ 2-Round Biotin-aRNA Amplification Kit (Epicentre). The amplified RNA was then analyzed for gene expression using Illumina Human HT-12 expression v4 BeadChips. Pre-hybridization treatment, hybridization and post-hybridization washes were performed using the Illumina whole-genome gene expression direct hybridization assay (Illumina, Manual 11322187A).

### Microarray Data Analysis

Data pre-processing: Basic Illumina chip data and experimental quality analyses were performed using the GenomeStudio software V2011.1. For probe summarization and data normalization the Robust Multi-array Analysis (RMA) method was used as described previously^40^.

Data were then filtrated in several steps, i.e. first, probe sets not having detection p-values of less than 0.01 in at least 80% of the samples were excluded, followed by re-annotation using the R package IlluminaHumanv4.db^41^. Probe sets with no or expired annotations were then removed. Finally, signals were log 2 transformed.

Next, differentially expressed genes were determined using LIMMA for R^42^ using a FDR cutoff of 5%. The resulting profiles were z-score normalized and clustered using the k-means algorithm to give 16 partitions. Each cluster was tested for enriched GO terms using DAVID^43^. Heatmaps were created in Genesis^44^ using hierarchical clustering using the correlation distance (1-*r*) as a measure of similarity.

### Data availability

The datasets generated during the current study have been deposited in NCBI’s Gene Expression Omnibus and are accessible through GEO Series accession number GSE101388 (https://www.ncbi.nlm.nih.gov/geo/query/acc.cgi?acc=GSE101388).

## Electronic supplementary material

Supplementary Information
