# Supplementary material for: Human Primary Bone Marrow Mesenchymal Stromal Cells and Their in vitro Progenies Display Distinct Transcriptional Profile Signatures
Source: Sci Rep. 2017 Sep 4;7:10338. doi: 10.1038/s41598-017-09449-x (PMC5583257; doi:10.1038/s41598-017-09449-x)
Supplement: Supplementary file 1 — Supplementary Information [file 41598_2017_9449_MOESM1_ESM.pdf]

Human Primary Bone Marrow Mesenchymal Stromal Cells and Their *in vitro* Progenies Display Distinct Transcriptional Profile Signatures

Roshanak Ghazanfari,[1,2] Dimitra Zacharaki,[1,2] Hongzhe Li,[1,2] Hooi Ching Lim,[1,2] Shamit Soneji,[2] and Stefan Scheding [1,2,3]

[1] Lund Stem Cell Center, University of Lund, Lund, Sweden

[2] Division of Molecular Hematology, Department of Laboratory Medicine, University of Lund, Lund, Sweden

[3] Department of Hematology, Skåne University Hospital Lund, Sweden

Supplementary Table S1: List of the genes in clusters 3 and 5*

Cluster 3

CTSC	TNFRSF1B	STOM	APBB1IP	HLA-E	FOSB	PSME1	SLC7A2
APOL3	HLA-DRB6	ZFP36	GLDN	ECGF1	AIM1	HLA-DRB1	PAIP2
MRPS31	PSAP	BST2	NUP88	C7orf11	LIPA	EXOSC10	CCDC130
CSTF2	TBP	SNRNP40	APOE	HLA-B	SDHA	HNRNPF	CYBA
HLA-F	B2M	PARP12	HIST2H2AC	NXF1	ITM2B	LARP4	PSMB9
NUP62	SLC44A1	C1QTNF1	OLFML2B	IFI16	DUSP1	PSMB8	HMGB3
PPT1	EIF2B5	FAU	CITED4	GTPBP6	TMEM218	EIF4A3	PTPRA
HLA-H	NOP58	FOS	STK11IP	FLJ37786	NDRG1	SERPING1	EML3
OR6C76	MFSD1	RFX5	HLX	MAPKAPK2	PPM1G	HCP5	DDX39
WDR86	HACL1	DPP4	CD68	SEPP1	RGS2	FAM96A	MAPKAPK3
C22orf9	ARMET	C11orf73	NAV2	NPHS2	VCAM1	ZC3H11B	SNRPB
EGR1	PCID2	OAS2	PSME2	ALPL	GSDMD	SLC15A3	HLA-G
PDGFRA	PTK6	ADD3	PIM2	S1PR3	RHOBTB1	KCNJ3	FLJ42842
SMAP2	CDH7	LINGO4	UCHL5IP	CMTM7	SUPT7L	TMEM30A	HIST2H2AA4
LITRK4	GMDS	CTSC	TCEB3C	GRWD1	C19orf48	UNG	MIR658
TMEM69	HSDL2	PPARG	FABP5L2	PCCA	RIBC1	PTPN12	DNAJA1

Cluster 5

DKK3	CSRP1	COL8A1	LDLR	LIMS2	STC2	ASNS	POSTN	HOMER2	LOX
ITGA11	PDE7B	PSAT1	ASNS	COL12A1	TAGLN	KRT7	HAPLN1	LOXL1	LMCD1
COL1A2	DACT1	HAS1	MTHFD2	CRYAB	FLNC	COL5A2	CRABP2	DSTN	CRIP2
MFAP2	CDH2	CD151	SDC2	CNN1	SLC1A5	WSB2	PPP1R3C	PLAA	IARS
COL1A1	DSTN	FOXD1	THBS1	AFAP1	DACT1	CKB	CARS	THBS3	B4GALT2
COL4A1	CALD1	KIAA164	TRIB3	NUPR1	MFAP5	MYL9	MTHFD2	HAPLN1	PYCR1
NDUFA10	DAG1	C11orf41	MTCH1	MYL6	FRMD6	TUBB2B	LBH	HSPB7	RAB23
MYL6	PALM	MID1	SLC16A5	POSTN	TAGLN	CBS	C16orf7	HCFC1R1	FLRT2
COL5A1	SPARC	PCBD1	DPYSL3	TGDS	SYT11	C9orf3	PHGDH	FARP1	ZFPL1
KIAA0363	BEND6	PCBP4	PEA15	SH3BP4	FRMD6	LIMA1	BCAR3	CHST14	ENAH
CLTB	TULP4	MLH3	SLC2A1	PTPRF	ALPK2	NUAK1	LOXL3	PALLD	TPM1
ACTB	LYPD1	HCFC1R1	MAP1A	FAM69A	LARGE	ANXA5	CARS	RIMS3	ARHGDIA
C5orf23	TMEM90B	TNS1	TUFT1	C1orf198	PCK2	FER1L3	KCNK2	LRSAM1	FAT1
EFS	ATF4	EIF1	TP53I11	COMP	LMOD1	EHD2	SLIT3	TRNP1	TPM1
EVL	VLDLR	ANXA2P1	GALNT10	TSPO	TBC1D20	RUSC2	FLJ35258	DACT3	KCTD15
GPER	TBCB	GREM1	KLHL9	FKBP9L	ESYT1	RNH1	UBE2D4	SLC6A8	MYLK
SLC38A2	CBR3	CPA4	MYOF	RAB23	ITGBL1	NFE2L1	NDUFB7	PDLIM1	ADCY4
LRRC15	FNDC1	SNTA1	CYB5R3	COPZ2	FLNA	CNTNAP1	C2orf24	FBXL7	MATN2
CABLES1	KRT81	HAS3	LARGE	UHRF1BP1	LRRC32	MGC42630	MGC102966	SCARF2	LYPD6B
ATXN2	TMEM184B	TNK2	ANXA2P1	COL4A2	NTN4	FBXL5	TBC1D20	JUB	CDC14A
NTM	GCC1	HSPB2	GDPD1	FNDC1	POFUT2	S100A11	C5orf53	UNC5B	KIFAP3
LHPP	SLC7A1	ACLY	SCRG1	SLC7A5	ZFHX3	PIP3-E	KRT78	POFUT2	INHBE
MYLK	FAM180A	SPTAN1	ARL2	TPM2	STBD1	NTM	RNF150	ATP6V0E2	MEX3B
SYTL2	PENK	TPD52L1	PTN	FKSG30	NAV1	ECM1	PFKP	TMUB1	P4HA3
B3GALT6	PCK2	C1QTNF5	P2RX6	SEMA4F	CRLF1	TMEM166	SMPD1	PTPRF	ABLIM1
CXXC5	MIR889	PPP1R14B	CALD1	BCAS4	GLS	GDI1	PIP5K1C	PDGFC	FUBP3
GALNT1	FSTL3	MCAM	KIAA0427	ID3	DYNLRB1	GPR1	C14orf78	HSPB8	SLC3A2
ID2	COL8A2	LEPRE1	ATOH8	SC4MOL	RAP1B	BAMBI	CDC42EP1	CXCR7	ST8SIA4
COL8A2	LIMCH1	PXDN	C20orf30	RBCK1	CAP2	THY1	EYA2	RHOBTB1	RTN4
TCEA2	WARS	LSS	IGFBP7	RAB11FIP2	TTC3	ZCCHC14	TPD52L1	PFN1	BOK
FAM171A1	KCNMA1	PARVA	DAB2	LMF1	SIPA1L1	WARS	CREB3L2	FBXW4	MGC72080
FLJ40504	CTGF	ALCAM	ENC1	VCL	CTGF	CYB5R3	COL16A1	MYH9	CNN2
KCTD11	CSPG4	MYH9	SLC1A4	C5orf13	MET	TMEM47	PRSS23	ADM2	NLRX1
SCAND2	GRPEL2	SOX4	SEC61A1	BGN	NUAK1	PLS3	ISLR	EIF4EBP1	CLSTN1
PPAP2A	YIF1B	DCI	C18orf1	FZD2	C7orf50	IL11	ZNF197	C6orf48	NAGK
LAYN	ABI3BP	SORBS2	SASH1	SIRPA	TP53INP2				

* Genes are indicated based on official gene symbol in NCBI

Supplementary Table S2: List of the primers used for Real-Time PCR

Name	Forward	Reverse	Product size (bp)
RPL11	AAAGGTGCGGGAGTATGAGTT	TCCAGGCCGTAGATACCAATG	126
OAS2	ACGTGACATCCTCGATAAAACTG	GAACCCATCAAGGGACTTCTG	100
FOSB	ACCCTCTGCCGAGTCTCAAT	GAAGGAACCGGGCATTTC	109
SERPING1	TGCCCATGATGAATAGCAAG	TTCTGGGGTACCAGGATCAC	121
ALPL	AGCTGAACAGGAACAACGTG	CAGCAAGAAGAAGCCTTTGG	98
PPARG	TGCAGGTGATCAAGAAGACG	GAAGGGAAATGTTGGCAGTG	112

Supplementary Figure S1

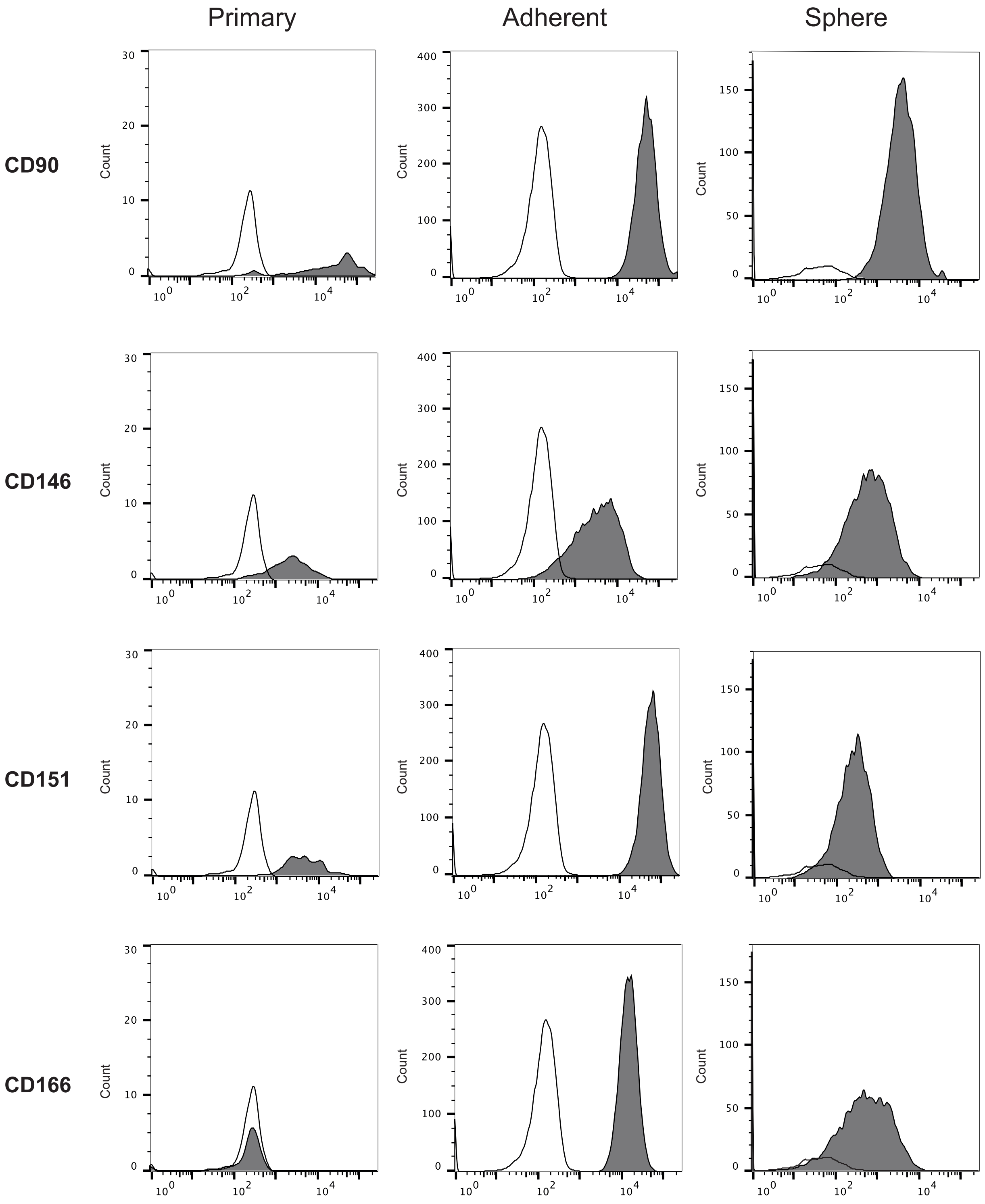

Supplementary Figure S1. Freshly isolated primary, and adherent and non-adherent (sphere) culture-expanded BM-MSCs (passage 3) were stained with antibodies against CD90, CD146, CD151 and CD166. A representative set of surface marker histograms (log scale) from a total of three independent experiments are shown in closed histograms. Corresponding isotype controls are indicated in open histograms.
